# Antibiotic usage in surgical prophylaxis: surveillance of clean and clean-contaminated surgeries in a tertiary care hospital

**DOI:** 10.3389/fpubh.2026.1759017

**Published:** 2026-05-04

**Authors:** Geetha Kandasamy, Vijayakumar Arumugam, Khalid Orayj, Mona Almanasef, Rayah Asiri, Asma M. Alshahrani, Amjad Hmlan, Hanan Alshareef, Kavitha Karunakaran

**Affiliations:** 1 Department of Clinical Pharmacy, College of Pharmacy, King Khalid University, Abha, Saudi Arabia; 2 Head Pharmacy Services, Kovai Medical Center and Hospital (KMCH), Coimbatore, India; 3 Department of Clinical Pharmacy, College of Pharmacy, Shaqra University, Dawadimi, Saudi Arabia; 4 Department of Pharmacy Practice, Faculty of Pharmacy, University of Tabuk, Tabuk, Saudi Arabia; 5 Department of Pharmacy Practice, KMCH College of Pharmacy, Coimbatore, India

**Keywords:** adherence, antimicrobial stewardship, clean surgery, clean-contaminated surgery, guideline adherence, surgical antibiotic prophylaxis

## Abstract

**Background:**

Surgical antibiotic prophylaxis (SAP) is an essential part of perioperative care, yet adherence to recommended practices varies across surgical categories. Differences in antibiotic choice and duration between clean and clean-contaminated procedures highlight the need to understand local prescribing patterns. This study aimed to describe SAP practices in a tertiary care hospital and assess adherence to institutional SAP guidelines in clean and clean-contaminated surgeries.

**Methods:**

A prospective observational cross-sectional study was conducted over 6 months in a tertiary care hospital. Adult patients undergoing elective clean or clean-contaminated surgeries were included. Information on perioperative antibiotic use was collected from medical records, and adherence to institutional prophylaxis guidelines was assessed. Data were analyzed using descriptive statistics, Chi-square or Fisher's exact tests, *t*-tests, relative risks, odds ratios, and multivariate logistic regression. A two-tailed *p* < 0.05 was considered statistically significant.

**Results:**

A total of 170 patients were assessed, including 143 clean and 27 clean-contaminated surgical cases. Significant differences were observed in antibiotic selection between the two categories; cefuroxime was used more frequently in clean surgeries (50.3%) than in clean-contaminated procedures (25.9%), with a relative risk of 1.94 (95% CI 1.00–3.78; *p* = 0.037). Clean surgeries were associated with higher adherence to guideline-recommended prophylaxis, particularly for appropriate antibiotic choice (83.2 vs. 63.0%; *p* = 0.032) and appropriate duration (90.2 vs. 66.7%; *p* = 0.003). Overall appropriateness, measured using the Antibiotic Prophylaxis Appropriateness Index (APAI), was higher in clean surgeries (89.4%) compared with clean-contaminated procedures (76.3%; *z* = 2.98; *p* = 0.003). In multivariate analysis, clean-contaminated surgery (adjusted OR 3.89; 95% CI 1.37–11.05; *p* = 0.009) and prolonged prophylaxis duration (adjusted OR 4.61; 95% CI 1.74–12.18; *p* = 0.003) were independently associated with non-adherence. Surgical site infections were infrequent, with two cases (1.4%) reported in clean surgeries and none following clean-contaminated procedures.

**Conclusion:**

Antibiotic prophylaxis practices varied between clean and clean-contaminated surgeries, with higher adherence observed in clean procedures, particularly in antibiotic selection and duration. Prolonged prophylaxis was more common in clean-contaminated surgeries and was associated with non-adherence. Given the observational design, causal inferences cannot be established. These findings highlight potential targets for improving guideline-based prophylaxis through antimicrobial stewardship strategies.

## 1 Introduction

Surgical antibiotic prophylaxis (SAP) is an important strategy for reducing the risk of postoperative surgical site infections (SSIs) by administering a short course of antimicrobial agents prior to surgical incision (1). SSIs remain among the most common healthcare-associated infections and represent a major postoperative complication (2), contributing significantly to increased morbidity, mortality, and healthcare expenditure worldwide (3).

Evidence suggests that appropriate SAP is associated with a reduction in SSI risk (4). Key elements include appropriate clinical indication, selection of an appropriate antimicrobial agent covering likely pathogens, and achieving adequate bactericidal concentrations at the surgical site during the period of potential contamination (4, 5). Despite well-established recommendations, considerable variability in SAP adherence persists across healthcare institutions (6). While indication and dosage adherence are generally acceptable, inappropriate antimicrobial choice, timing, and extended duration remain frequent challenges (6, 7). SSIs continue to pose a substantial burden of morbidity, particularly in low- and middle-income countries (8). SAP is intended to achieve adequate antimicrobial tissue concentrations prior to incision and is associated with reduced SSI risk (9, 10).

International guidelines, including those from the American Society of Health-System Pharmacists (ASHP), emphasize evidence-based SAP practices to minimize postoperative infections (11–15). These guidelines recommend antibiotic administration within 60 min of incision, weight-based dose adjustments, and repeat dosing for prolonged procedures (16–19). A single postoperative dose or discontinuation within 24 h is advised to minimize unnecessary antimicrobial exposure (16). In addition to ASHP recommendations, both the World Health Organization (WHO) and the Centers for Disease Control and Prevention (CDC) emphasize timely pre-incision dosing, appropriate agent selection, and limiting postoperative antimicrobial use to prevent resistance and reduce SSI burden, reinforcing the global necessity for adherence to evidence-based prophylaxis protocols (20, 21). SSIs significantly increase morbidity, mortality, hospital stay duration, and treatment costs (22). An SSI is defined as an infection occurring within 30 days of surgery, or within 90 days when prosthetic material is implanted (23). Globally, approximately 11.2 SSIs occur per 100 surgical procedures (24), and in developing settings, around 10% of hospitalized patients acquire healthcare-associated infections, with SSIs accounting for 5.6% of cases (23). In an Indian prospective study, the incidence of SSIs was reported as 3.7% in clean surgeries and 13.04% in clean-contaminated procedures, highlighting the ongoing burden of postoperative infections in the country despite established preventive guidelines (25). The present investigation was carried out in a tertiary-level teaching hospital in India, serving a large and varied patient population and performing both elective and emergency surgeries, making it a relevant setting to assess SAP practices in routine clinical care.

SAP is intended to reduce bacterial load at the incision site and support host defense mechanisms against infection (26). However, despite proven benefits, misuse of prophylactic antibiotics remains widespread (27), occurring in up to 25%−50% of elective surgeries (28). Antibiotic prophylaxis should therefore be reserved for cases where benefits outweigh risks (29). Overuse, particularly of broad-spectrum agents, contributes to antibiotic resistance (29), whereas shorter SAP courses help reduce toxicity, cost, and resistance development (30).

The escalating threat of antimicrobial resistance has prompted global antimicrobial stewardship initiatives to promote rational antibiotic use and limit unnecessary perioperative exposure (31). Nonetheless, adherence to guideline-directed SAP remains suboptimal in routine clinical practice (32), particularly in resource-constrained settings, highlighting the need for ongoing surveillance and quality improvement efforts.

Surgical wound classification guides antimicrobial prophylaxis decision-making. Clean operations involve sterile surgical fields without entry into the respiratory, gastrointestinal, genitourinary, or biliary tracts, with strict aseptic technique and no inflammation. Examples include thyroidectomy, mastectomy, hernia repair, orthopedic implant surgery, and neurosurgical procedures (15, 20). Clean-contaminated procedures involve controlled entry into these tracts without significant contamination, such as cholecystectomy, gastrectomy, bowel resection, non-perforated appendectomy, cesarean delivery, hysterectomy, and urologic surgery (15, 20).

Limited prospective surveillance studies have evaluated adherence to surgical antibiotic prophylaxis (SAP) guidelines in clean and clean-contaminated surgeries in tertiary-care settings. This study provides novel prospective evidence by systematically assessing multiple dimensions of SAP adherence, including antibiotic selection, timing, dosing, and duration, in both clean and clean-contaminated surgeries in a tertiary care teaching hospital in this region. Understanding deviations from evidence-based SAP recommendations is essential for guiding targeted antimicrobial stewardship interventions and improving perioperative prescribing practices. This prospective observational study evaluated real-world antibiotic prophylaxis practices and adherence to institutional SAP guidelines in routine clinical care, without implying causal relationships. Improving adherence to evidence-based SAP practices may support antimicrobial stewardship efforts, potentially contributing to reduced antimicrobial resistance and improved healthcare resource utilization (33). Therefore, this study aimed to describe antibiotic prophylaxis practices in clean and clean-contaminated surgeries and assess adherence to institutional SAP guidelines in a tertiary care hospital.

## 2 Methods

### 2.1 Study design and setting

A prospective observational cross-sectional study was conducted from January 2016 to June 2016 in the surgical departments of Kovai Medical Center and Hospital (KMCH), a tertiary care teaching hospital in Coimbatore, Tamil Nadu, India. The study aimed to describe antibiotic usage patterns and adherence with institutional surgical prophylaxis guidelines among patients undergoing clean and clean-contaminated surgical procedures. The study findings are limited to describing associations and do not imply causal relationships between variables or outcomes. The study framework included adult patients (≥18 years) undergoing elective clean and clean-contaminated surgeries. Perioperative surgical antibiotic prophylaxis practices were evaluated as the exposure of interest, with comparison between adherence and non-adherence to institutional guidelines. Outcomes included appropriateness of antibiotic selection, dose, timing, and duration, assessed individually and as a composite measure using the Antibiotic Prophylaxis Appropriateness Index (APAI).

### 2.2 Study population

The study population comprised adult patients (≥18 years) undergoing elective clean and clean-contaminated surgical procedures during the 6-month study period at the study site. A total of 170 eligible patients were included, comprising 143 clean and 27 clean-contaminated procedures. Patients were excluded if they underwent emergency surgeries, had contaminated or dirty wounds, were receiving therapeutic antibiotics prior to surgery, or had incomplete clinical documentation. Cases with documented contraindications to standard prophylactic antibiotics were also excluded where applicable.

### 2.3 Data collection

A structured data collection form was used to capture patient characteristics (age and gender), type of surgery, and perioperative antibiotic prophylaxis details. Information on the antibiotic agent administered, dosage, and duration was obtained from patient medical records and surgical case notes. Adherence with institutional antibiotic prophylaxis guidelines was evaluated based on three components: appropriate antibiotic selection, appropriate dosing, and appropriate duration, defined as discontinuation within 24 h after surgery. These components were combined to derive the APAI to assess overall adherence within the study setting. Data were extracted solely from medical records and perioperative documentation.

#### 2.3.1 Institutional surgical antibiotic prophylaxis guidelines (gold standard)

The gold standard for assessing adherence was based on institutional surgical antibiotic prophylaxis guidelines developed by the hospital's Infection Control Committee as part of the antimicrobial stewardship program. These guidelines were formulated based on the institutional antibiogram and local antimicrobial susceptibility patterns. They provided recommendations for antibiotic selection according to surgical category (clean and clean-contaminated procedures), as well as guidance on dosing, timing of administration, and duration of prophylaxis, including discontinuation within 24 h postoperatively.

The guidelines were reviewed by infection control specialists, clinical pharmacists, and surgical representatives prior to implementation and were disseminated across surgical departments through institutional protocol documents and Infection Control Committee communications. They were accessible to all prescribing clinicians *via* hospital systems and the institutional formulary throughout the study period. It is important to note that these guidelines served as the institutional standard for practice and evaluation; however, the study itself assessed adherence to these predefined recommendations in a real-world clinical setting.

### 2.4 Statistical analysis

Data were entered into Microsoft Excel and analyzed using IBM SPSS Statistics version 18. Categorical variables were expressed as frequencies and percentages, while continuous variables were reported as mean ± standard deviation (SD). Comparisons between clean and clean-contaminated surgeries were performed using the Chi-square test or Fisher's exact test for categorical data and independent-samples *t*-tests for continuous variables. To examine factors associated with adherence to surgical antibiotic prophylaxis, relative risks (RRs) and odds ratios (ORs) with 95% confidence intervals were calculated. Variables showing a *p*-value < 0.10 in univariate testing were included in a multivariate logistic regression model. Given the relatively small number of clean-contaminated cases, subgroup analyses were exploratory in nature, and findings should be interpreted cautiously due to limited statistical power and potential model instability. Model fit was assessed using the omnibus chi-square test, Nagelkerke *R*^2^, and Hosmer–Lemeshow goodness-of-fit test. Multicollinearity was assessed using variance inflation factor (VIF), with values < 2 considered acceptable. A two-sided *p*-value < 0.05 was considered statistically significant.

### 2.5 Ethical considerations

Ethical approval was obtained from the Institutional Ethics Committee of KMCH (EC/AP/450/03/2016), and written informed consent was obtained from all participants after explaining the study's purpose and procedures. Patient confidentiality was ensured by anonymizing all data and excluding personal identifiers, in accordance with institutional and national ethical guidelines.

## 3 Results

A total of 170 patients were included in the study, comprising 143 clean surgeries and 27 clean-contaminated surgeries. The demographic and surgical characteristics of both groups are presented in Table 1. Gender distribution did not significantly differ between the groups (*p* = 0.35). Males accounted for 65.0% of the clean-surgery group and 74.1% of the clean-contaminated group. Age categories were also similarly distributed (*p* = 0.42). The largest proportion of patients in both groups fell within the 51–70-year age range. The mean age was comparable between clean (48.9 ± 15.0 years) and clean-contaminated surgeries (51.3 ± 14.8 years), with no significant difference observed (*p* = 0.44). A significant variation was noted in the departmental distribution of surgeries (*p* = 0.016). Clean surgeries were more frequently performed in neurosurgery (33.6%) and general surgery (31.5%), whereas clean-contaminated procedures were more common in ENT (37.0%) and cardiac surgery (33.3%). Surgical site infections were rare, with two cases (1.4%) observed in the clean surgery group and none in clean-contaminated procedures (*p* = 0.351). Although the low SSI rate was observed, it should be interpreted with caution due to the small sample size.

**Table 1:** Demographic and surgical characteristics by surgery category.

Variable	Clean (*n* = 143)	Clean-contaminated (*n* = 27)	χ^2^/Fisher *p*
Gender			
Male	93 (65.0%)	20 (74.1%)	0.35
Female	50 (35.0%)	7 (25.9%)	
**Age group (years)**			0.42
< 30	25 (17.5%)	5 (18.5%)	
31–50	50 (35.0%)	5 (18.5%)	
51–70	58 (40.6%)	14 (51.9%)	
>70	10 (7.0%)	3 (11.1%)	
**Mean** **±SD age (yrs)**	48.9 ± 15.0	51.3 ± 14.8	0.44 (*t*-test)
**Department**			**0.016**
General	45 (31.5%)	6 (22.2%)	
Neurosurgery	48 (33.6%)	5 (18.5%)	
Cardiac	29 (20.3%)	9 (33.3%)	
ENT	13 (9.1%)	10 (37.0%)	
Pediatric	4 (2.8%)	1 (3.7%)	
Surgical site infection	2 (1.4%)	0 (0.0%)	0.351

SD, standard deviation. Bold values indicate statistically significant results (*p* < 0.05).

Table 2 presents the pattern of prophylactic antibiotic use across clean and clean-contaminated surgeries. Notable differences were observed in the choice of antibiotics between the two categories. Cefuroxime was the most commonly used agent overall and was administered significantly more often in clean surgeries (50.3%) compared with clean-contaminated surgeries (25.9%). This difference was statistically significant (RR 1.94, 95% CI 1.00–3.78; *p* = 0.037). In contrast, the use of amoxicillin–clavulanate was higher in clean-contaminated procedures (22.2%) than in clean surgeries (10.5%), although this difference did not reach statistical significance (*p* = 0.11). Similar patterns were observed for cefoperazone–sulbactam, ceftriaxone, and cefoperazone alone, all of which showed higher utilization rates in clean-contaminated surgeries, but without significant differences between groups. Several antibiotics such as cefotaxime, cefazolin, ofloxacin, gentamicin, and ciprofloxacin were used exclusively in clean surgeries, though their overall use was minimal and the differences were not statistically meaningful (all *p* > 0.58). Piperacillin–tazobactam use was rare, recorded in only one clean-surgery case. The use of third- or fourth-generation cephalosporins was similar between the two groups (18.9 vs. 22.2%; RR 0.85, 95% CI 0.38–1.91; *p* = 0.72), indicating no significant difference in the preference for higher-generation cephalosporins across surgery categories.

**Table 2:** Antibiotic utilization patterns by surgery category.

Antibiotic	Clean (*n* = 143) *n* (%)	Clean-contaminated (*n* = 27) *n* (%)	RR (95% CI)	Fisher *p*
Cefuroxime	72 (50.3)	7 (25.9)	1.94 (1.00–3.78)	**0.037**
Amoxicillin-clavulanate	15 (10.5)	6 (22.2)	0.47 (0.20–1.13)	0.11
Cefoperazone-sulbactam	6 (4.2)	2 (7.4)	0.57 (0.13–2.51)	0.61
Ceftriaxone	5 (3.5)	2 (7.4)	0.47 (0.10–2.20)	0.32
Cefoperazone (alone)	4 (2.8)	2 (7.4)	0.38 (0.08–1.85)	0.22
Cefotaxime	5 (3.5)	0 (0.0)	–	0.58
Cefazolin	5 (3.5)	0 (0.0)	–	0.58
Ofloxacin	3 (2.1)	0 (0.0)	–	0.61
Gentamicin	2 (1.4)	0 (0.0)	–	0.68
Ciprofloxacin	2 (1.4)	0 (0.0)	–	0.68
Piperacillin-tazobactam	1 (0.7)	0 (0.0)	–	0.77
*Any 3rd/4th-gen cephalosporin*	27 (18.9)	6 (22.2)	0.85 (0.38–1.91)	0.72

RR, relative risk; CI, confidence interval; n, number of patients; Fisher p, *p*-value derived from Fisher's exact test. Bold values indicate statistically significant results (*p* < 0.05).

Adherence with key components of antibiotic prophylaxis is summarized in Table 3. Overall adherence was high in both surgery categories, although notable differences were observed for specific parameters. Appropriate antibiotic selection was achieved in 83.2% of clean surgeries compared with 63.0% of clean-contaminated surgeries. This difference was statistically significant (*p* = 0.032), with clean surgeries showing higher adherence (RR 1.32; 95% CI 0.98–1.78; OR 2.92; 95% CI 1.19–7.14). Adherence with appropriate dosing was comparable between groups, with 94.4% in clean surgeries and 88.9% in clean-contaminated surgeries. This difference was not statistically significant (*p* = 0.38). Similarly, adherence to appropriate timing of prophylaxis was high in both groups 93.7% in clean and 92.6% in clean-contaminated procedures with no meaningful difference (*p* = 0.69). However, a significant variation was noted in duration of prophylaxis. Appropriate duration was followed in 90.2% of clean surgeries but only 66.7% of clean-contaminated surgeries. This difference was statistically significant (*p* = 0.003), with a higher likelihood of appropriate duration among clean surgeries (RR 1.35; 95% CI 1.03–1.78; OR 4.61; 95% CI 1.74–12.18).

**Table 3:** Adherence with antibiotic prophylaxis guidelines by surgical category.

Parameter	Clean (*n* = 143)	Clean-contaminated (*n* = 27)	RR (95% CI)	OR (95% CI)	Fisher *p*
Appropriate antibiotic	119 (83.2)	17 (63.0)	1.32 (0.98–1.78)	2.92 (1.19–7.14)	**0.032**
Appropriate dose	135 (94.4)	24 (88.9)	1.06 (0.92–1.22)	2.11 (0.52–8.52)	0.38
Appropriate timing	134 (93.7)	25 (92.6)	1.01 (0.90–1.14)	1.19 (0.24–5.84)	0.69
Appropriate duration	129 (90.2)	18 (66.7)	1.35 (1.03–1.78)	4.61 (1.74–12.18)	**0.003**

RR, relative risk; OR, odds ratio; CI, confidence interval; n, number of patients; Fisher p, *p*-value derived from Fisher's exact test. Bold values indicate statistically significant results (*p* < 0.05).

The Comprehensive APAI assessed overall adherence to recommended prophylaxis standards by integrating four weighted components: appropriate agent, dose, timing, and duration. The comparative APAI performance for clean and clean-contaminated surgeries is summarized in Table 4. The appropriate antibiotic component contributed the largest weight (35%) to the index. Clean surgeries demonstrated significantly higher adherence (83.2%; 95% CI: 76.3–89.1) than clean-contaminated procedures (63.0%; 95% CI: 43.2–80.5). This translated into a 7-point difference in weighted scores (29.1 vs. 22.1), which was statistically significant (*z* = 2.19; *p* = 0.028). Adherence with the appropriate dose was high in both categories, with weighted scores of 18.9 for clean and 17.8 for clean-contaminated surgeries. The difference was small (1.1 points) and not statistically significant (*p* = 0.37). Similarly, the appropriate timing of prophylaxis demonstrated nearly identical adherence between the two groups. Weighted scores were 23.4 for clean and 23.1 for clean-contaminated surgeries, with no meaningful difference (*p* = 0.86). A substantial difference was observed in the appropriate duration component, where adherence was 90.2% (95% CI: 84.0–94.8) for clean surgeries compared with 66.7% (95% CI: 46.0–83.5) for clean-contaminated procedures. This resulted in a 4.7-point difference in weighted scores, which was statistically significant (*z* = 2.52; *p* = 0.012). When all components were integrated into the overall APAI score, clean surgeries achieved a significantly higher mean index value (89.4% ± 2.3 SE) compared with clean-contaminated surgeries (76.3% ± 4.8 SE). The overall difference of 13.1 points was statistically significant (*z* = 2.98; *p* = 0.003), indicating higher overall prophylaxis appropriateness scores in clean procedures.

**Table 4:** Comprehensive antibiotic prophylaxis appropriateness index (APAI).

Component	Weight (%)	Clean % (95% CI)	Clean-cont. % (95% CI)	Weighted score clean	Weighted score CC	Difference	*z*-score	*p*-value
Appropriate antibiotic	35%	83.2% (76.3–89.1)	63.0% (43.2–80.5)	29.1	22.1	7.0	2.19	**0.028**
Appropriate dose	20%	94.4% (89.2–98.1)	88.9% (70.8–97.6)	18.9	17.8	1.1	0.89	0.37
Appropriate timing	25%	93.7% (88.3–97.2)	92.6% (75.7–99.1)	23.4	23.1	0.3	0.18	0.86
Appropriate duration	20%	90.2% (84.0–94.8)	66.7% (46.0–83.5)	18.0	13.3	4.7	2.52	**0.012**
Total APAI score	100%	89.4% (±2.3 SE)	76.3% (±4.8 SE)	—	—	13.1	2.98	0.003

CC, clean-contaminated surgery; CI, confidence interval; SE, standard error; z-score: standardized test statistic used for comparison between groups. Bold values indicate statistically significant results (*p* < 0.05).

The duration of antibiotic prophylaxis differed significantly between clean and clean-contaminated surgeries (Table 5). Differences in duration of antibiotic prophylaxis were observed between clean and clean-contaminated surgeries. The majority of clean procedures appropriately received prophylaxis for ≤ 24 h (82.5%), whereas only 59.3% of clean-contaminated surgeries adhered to this recommended duration. Conversely, prolonged antibiotic use beyond 24 h was notably higher among clean-contaminated surgeries compared with clean procedures (40.7 vs. 17.5%). This difference was statistically significant (*p* = 0.0014). Mean duration of prophylaxis was 23.1 ± 9.4 h in clean surgeries and 38.4 ± 15.2 h in clean-contaminated surgeries (*p* < 0.001). The median duration was 22 h (IQR: 18–26) and 36 h (IQR: 28–48), respectively. Mean deviation from the recommended 24-h duration was −0.9 ± 9.4 h in clean surgeries and +14.4 ± 15.2 h in clean-contaminated surgeries (*p* < 0.001).

**Table 5:** Duration of antibiotic prophylaxis by surgical category.

Duration	Clean *n* (%)	Clean-contaminated *n* (%)	*p*
≤ 24 h (prophylactic)	118 (82.5)	16 (59.3)	**0.0014**
>24 h (therapeutic)	25 (17.5)	11 (40.7)	
Mean duration (h) Mean ± SD	23.1 ± 9.4	38.4 ± 15.2	< 0.001
Median duration (h) IQR	22 (18–26)	36 (28–48)	—
Mean deviation from 24 h (h) Mean ± SD	−0.9 ± 9.4	+14.4 ± 15.2	< 0.001

SD, standard deviation; IQR, inter quartile range. Bold values indicate statistically significant results (*p* < 0.05).

A multivariate logistic regression model was constructed to identify factors associated with non-adherence to antibiotic prophylaxis guidelines. The analysis revealed that certain variables were independently associated with a higher likelihood of non-adherence (Table 6). Clean-contaminated surgery was significantly associated with non-adherence, with nearly fourfold higher odds compared with clean procedures (adjusted OR 3.89, 95% CI 1.37–11.05; *p* = 0.009). The predicted probability of non-adherence in this group ranged between 41 and 48%. Inappropriate duration of prophylaxis showed the strongest association with non-adherence in the adjusted model. Patients receiving an inappropriate duration had over four times higher odds of non-adherence (adjusted OR 4.61, 95% CI 1.74–12.18; *p* = 0.003), with predicted probabilities between 55 and 65%. Other factors including age >60 years, male gender, use of broad-spectrum antibiotics, and multi-antibiotic regimens showed elevated odds ratios but did not reach statistical significance (all *p* > 0.05). Multi-antibiotic regimens demonstrated a borderline trend toward increased risk (OR 2.20, *p* = 0.09), but without statistical significance. Overall, the model suggests surgery type and inappropriate duration of prophylaxis as the key determinants of non-adherence in this cohort.

**Table 6:** Risk matrix for non-adherence (multivariate probability model).

Risk factor	Adjusted OR (95% CI)	β coefficient (SE)	Predicted probability (%)	Wald χ^2^	*p*-value
Clean-contaminated surgery	3.89 (1.37–11.05)	1.36 (0.51)	41%−48%	7.10	**0.009**
Age > 60 years	1.31 (0.56–3.04)	0.27 (0.44)	22%−27%	0.41	0.52
Male gender	1.12 (0.48–2.64)	0.11 (0.43)	18%−22%	**0.06**	**0.80**
Inappropriate duration	4.61 (1.74–12.18)	1.53 (0.52)	55%−65%	**8.67**	**0.003**
Broad-spectrum use	1.80 (0.78–3.92)	0.59 (0.44)	30%−35%	**1.76**	**0.18**
Multi-antibiotic regimen	2.20 (0.91–5.33)	0.79 (0.47)	32%−40%	**2.84**	**0.09**

Model χ^2^ (df = 6):14.82, *p* value: 0.021, Nagelkeree R^2^: 0.26, Hosmer–Lemeshow *p*: 0.64. Bold values indicate statistically significant results (*p* < 0.05).

## 4 Discussion

This study aimed to evaluate antibiotic prophylaxis practices in clean and clean-contaminated surgeries within a tertiary care hospital and to assess adherence to institutional surgical antibiotic prophylaxis guidelines. In this study, males comprised 65.0% of clean and 74.1% of clean-contaminated surgeries, consistent with earlier reports in which males predominated among surgical populations (34). Age patterns were also comparable, with most patients in the 51–70-year category in our cohort and the 41–60-year range in the previous study (34), highlighting the predominance of middle-aged and older adults in surgical case profiles.

Departmental distribution varied across settings. Clean surgeries were mainly performed in Neurosurgery and General Surgery, whereas clean-contaminated procedures were more common in ENT and Cardiac Surgery. In contrast, earlier studies reported Orthopedics as the leading category, followed by General Surgery, reflecting differences in institutional case mix and referral pathways (34).

Cefuroxime was the most frequently used prophylactic antibiotic in our cohort, particularly in clean surgeries (50.3%), aligning with earlier observations where third-generation cephalosporins were commonly prescribed (34, 35). Single-agent prophylaxis predominated in our study, consistent with Rehan et al. (36) who reported similar trends. The limited use of combination regimens such as amoxicillin–clavulanate and cefoperazone–sulbactam contrasts with previous studies where broader and multi-drug combinations were frequently used (34, 36). Higher-generation cephalosporin use in our setting remained lower than patterns of broad-spectrum prescribing documented elsewhere (34, 35).

Recent regional evidence further supports these observations. A study from Saudi Arabia reported that cefuroxime and ceftriaxone remained among the most prescribed agents for clean and clean-contaminated surgeries, with substantial variation in adherence to national protocols, particularly in clean-contaminated procedures (37). Similarly, an Ethiopian study evaluating SAP practices (38) found heavy reliance on broad-spectrum antibiotics and substantial deviations from guideline-recommended duration, reinforcing the need for stewardship an area in which our findings demonstrate comparatively better performance.

Adherence to prophylaxis guidelines in our study was generally high, especially in clean surgeries. Appropriate antibiotic selection (83.2%), dosing (94.4%), and timing (93.7%) were achieved at high rates, with duration being the most frequent area of deviation, particularly in clean-contaminated surgeries (66.7%). Inappropriate duration was strongly associated with non-adherence (Adjusted OR 4.61, *p* = 0.003). These findings are consistent with the report by Nalla Dilip et al. (39), who also observed higher rates of inappropriate duration in clean-contaminated wounds compared with clean procedures, concluding that duration remains the weakest component of adherence efforts in many institutions.

Surgical site infections were rare in our cohort, with two cases (1.4%) observed in clean surgeries. Although the small number of events limits robust comparisons, this low SSI rate is consistent with previous evidence suggesting an association between appropriate timing and dosing and lower infection risk. These findings align with international guidelines, including ASHP/IDSA recommendations, which emphasize timing and dosing as key determinants of SSI prevention (4, 20, 40, 41). Both SSI cases underwent culture and sensitivity testing, allowing targeted postoperative antibiotic therapy, which further supports antimicrobial stewardship by minimizing unnecessary broad-spectrum antibiotic use.

Compared with international data, adherence in our study was substantially higher. A previous study reported 89.4% inappropriate antibiotic selection (35), similar to high rates observed in Ethiopia (7) and Iran (42). Lower non-adherence levels were reported in Sudan (43), Qatar (44), and Nicaragua (45). In contrast, our study demonstrates comparatively stronger adherence across most prophylaxis parameters, with deviations largely limited to clean-contaminated procedures.

The findings of this study highlight areas within surgical antibiotic prophylaxis where adherence to institutional guidelines can be strengthened, particularly in clean-contaminated procedures. Variability in antibiotic selection and duration suggests that clearer communication of protocol expectations and more consistent application of recommended practices may support improved alignment with established standards.

These results may assist antimicrobial stewardship teams in identifying specific components of prophylaxis that require targeted oversight, such as duration of administration. Regular audits, enhanced prescriber education, and the integration of decision-support tools could help reinforce appropriate antibiotic use during the perioperative period. Emphasizing guideline-based practice may contribute to more rational antibiotic utilization, reduce unnecessary exposure to broad-spectrum agents, and support ongoing efforts to mitigate antimicrobial resistance within hospital settings. While the study does not evaluate clinical outcomes, the observed patterns of practice provide actionable insights for refining institutional policies and informing future quality-improvement initiatives in surgical care. Given the observational nature of the study, these findings should be interpreted as associations, and causal relationships cannot be inferred.

### 4.1 Strengths and limitations

This study offers valuable insights into real-world surgical antibiotic prophylaxis practices through prospective surveillance conducted in a high-volume tertiary care hospital. The use of a structured data collection process and a composite appropriateness index (APAI) enabled a comprehensive assessment of key components of surgical prophylaxis, including antibiotic selection, dose, timing, and duration. In addition, the inclusion of multiple surgical departments enhances the applicability of the findings across diverse perioperative settings.

However, certain limitations should be acknowledged. This was a single-center study, which may limit the generalizability of the findings to other healthcare settings with different institutional protocols and prescribing practices. Furthermore, there was a marked imbalance in the distribution of surgical categories, with a relatively small number of clean-contaminated procedures (*n* = 27) compared to clean procedures (*n* = 143), reflecting the case distribution during the study period. This unequal distribution may have reduced the statistical power for subgroup comparisons and may have affected the stability and reliability of the multivariate logistic regression analysis due to limited event numbers. Therefore, the results of subgroup analyses and regression modeling should be interpreted with caution.

In addition, although surgical site infections (SSIs) were observed during the study period, the observational design and absence of systematic long-term follow-up limited the ability to comprehensively evaluate the relationship between non-adherence to prophylaxis guidelines (e.g., prolonged antibiotic duration) and clinical outcomes, including SSIs and antimicrobial resistance patterns. Consequently, the clinical impact of guideline adherence could not be fully assessed. Finally, the cross-sectional design limits the ability to infer causal relationships between antibiotic prophylaxis practices and clinical outcomes.

### 4.2 Recommendations for future research

Future studies could further examine the factors that influence variation in antibiotic prophylaxis practices, particularly in clean-contaminated surgeries where lower adherence was observed. Understanding the clinical, operational, and behavioral reasons behind extended antibiotic use and inappropriate agent selection may guide more focused improvement strategies.

Research involving multiple institutions and a broader range of surgical specialties would help validate the patterns identified in this study and determine whether similar trends exist in different healthcare settings. Interventional studies assessing the impact of stewardship initiatives such as standardized order sets, digital prompts, or regular audit-and-feedback programs may also provide insights into approaches that can enhance adherence with prophylaxis guidelines.

Incorporating postoperative clinical outcomes, including surgical site infection rates and antimicrobial resistance profiles, in future investigations may offer a more comprehensive understanding of the consequences of inappropriate prophylaxis. Long-term surveillance or repeated cycle audits could further support continuous quality improvement and help refine institutional protocols over time.

## 5 Conclusion

This study identified differences in antibiotic prophylaxis practices between clean and clean-contaminated surgeries in a tertiary care setting. Overall adherence to institutional guidelines was generally satisfactory, with clean procedures showing higher adherence across key components, particularly antibiotic selection and duration. Prolonged prophylaxis was more frequently observed in clean-contaminated surgeries and was identified as a major contributor to non-adherence. The APAI scoring highlighted these variations, and multivariate analysis indicated that surgery type and inappropriate duration were significantly associated with inappropriate prophylaxis.

These observations highlight the relevance of structured and context-specific antimicrobial stewardship approaches. In this setting, electronic prescribing prompts integrated into hospital information systems may be considered to align antibiotic selection and duration with institutional guidelines at the point of care. Periodic audit-and-feedback mechanisms may provide opportunities to review prescribing patterns and support adherence to recommended practices. Regular clinician education and dissemination of updated protocols may also be incorporated as part of ongoing stewardship efforts.

## Data Availability

The original contributions presented in the study are included in the article/supplementary material, further inquiries can be directed to the corresponding author.
